# Organization of Medical Assistance in Poland for Ukrainian Citizens During the Russia-Ukraine War

**DOI:** 10.3389/fpubh.2022.904588

**Published:** 2022-07-07

**Authors:** Edyta Fatyga, Sylwia Dzięgielewska-Gęsiak, Małgorzata Muc-Wierzgoń

**Affiliations:** Department of Preventive Medicine, Medical University of Silesia in Katowice, Bytom, Poland

**Keywords:** armed actions, Ukrainian citizens, medical assistance, healthcare, Poland

## Abstract

From 24 February 2022 to 29 March 2022, Poland has taken 2,377,000 refugees fleeing Russia's invasion of Ukraine. They are mostly women, children, and the elderly. In this article, we present all activities and types of medical resources provided and organized in Poland for refugees from the first days of the conflict. Information has been compiled from the data available on the Polish Ministry of Health, other governmental and non-governmental organizations, foundations, and medical societies.

## Introduction

From 24 February 2022 to 29 March 2022, due to the attack of the Russian Federation on Ukraine, thousands of Ukrainian citizens began to travel to Poland in search of shelter and support. By the tenth week of the conflict, 2,377,000 refugees had crossed Poland's borders (1), mostly including women, children, and the elderly. Among them are people who need or will seek medical assistance.

Aim: We seek to identify characteristics of all activities and types of medical resources that are provided and organized in Poland for refugees from Ukraine territory affected by the war from the first days of the conflict.

## Methods

Online resources were systematically searched using the following terms: “Ukrainian refugees” and “Polish medical aid.” Information was obtained from the Polish Ministry of Health and other governmental and non-governmental organizations, foundations, and medical societies. This analysis was supplemented by a review of Polish law acts.

## Results

- Organizing medical points at Ukrainian-Polish border crossings and reception areas in each voivodeship.

In these areas, first aid is provided by doctors, nurses, paramedics, and volunteers.

- Government relased an act for assistance to Ukrainian citizens in connection with armed conflict in Ukrainian territory on 12 March 2022.^1^

The special act, in force from 24 February 2022, grants the right to medical benefits, reimbursement for drugs, and the supply of medical devices to Ukrainian citizens who came to Poland due to aggression from Russia on the same terms as for all insured Polish citizens (see text footnote 1). Refugees are also entitled to medicinal products under the health programs of the Ministry of Health and to services for the prevention and treatment of infectious diseases, i.e., vaccination against, tests for (antigen and PCR), and treatment related to COVID-19. Children are entitled to preventive vaccinations under the preventive vaccination calendar (Protective Vaccination Program—PSO for 2022). The act also provides free psychological assistance (financed from the state budget). The assistance will be provided by the commune head, mayor, or president of the city of the appropriate commune considering the place of residence of Ukraine citizens. People with disabilities from Ukraine were granted funds from the State Fund for the Rehabilitation of Disabled People.

- Preparation of leaflets written in Ukrainian for adult refugees from Ukraine about vaccinations against COVID-19 and about vaccinations for their children.

Vaccination of adults against COVID-19 in Ukraine began in February 2021, and the estimated vaccination rate for adults is ~35%.^2^ The vaccination status of children in Ukraine varies depending on the age group and region of the country, ranging from ~60 to 99%. Levels of vaccination against poliomyelitis, measles, diphtheria, tetanus, and pertussis are lower than in Poland. According to WHO data, in 2020 in Ukraine, the vaccination rate against measles was 81.9%, that against poliomyelitis was 84.2%, that against pertussis was 81.3%, and that against hepatitis was 80.9%.^3^,^4^

- Special medical transport (trains, ambulances, and air transport) for the injured/wounded because of military actions, young patients with cancer patients, patients in palliative care, pregnant women, women with toddlers, and children and/or orphans from Ukraine.

Each train can carry up to 160 patients, including 80 patients lying down. In such cases, medical assistance is provided by highly specialized medical centers. The National Hospital in Warsaw is prepared to receive the wounded. Moreover, 120 hospitals are deemed ready to accept the victims of the war.

- Organization of oncological treatment for young Ukrainian patients with cancer.

To coordinate the activities, a transfer center was established in Bocheniec—the “Jednorożec” clinic of Marian Wilemski (Unicorn Clinic). More than 500 Ukrainian children with cancer have been evacuated to a Unicorn Clinic. The facility adopts medical triage. Children and their families receive medical assistance and shelter there. Then, they are transported to specialized clinics in Poland and abroad (200 co-working hospitals in 28 countries). St. Jude Children's Research Hospital has set up a command center to help manage information and coordinate treatment worldwide.^5^

- Provision of targeted, highly specialized medical assistance, such as oncology, hematology, nephrology, diabetes, and neurology treatment, at departments and clinics in Poland.
- Launch of a 24-h crisis hotline dedicated to border services and staff of reception points accepting refugees from Ukraine to aid disabled people.^6^
- Proxy of the Minister of Health for transfer and continued treatment outside the Republic of Poland for Ukrainian patients staying in the territory of the Republic of Poland in connection with the ongoing war in the territory of Ukraine.
- Launch of the Teleplatform of First Contact with permanent medical service provided to Ukrainians.^7^
- Launch of a free helpline in Ukraine for people in need of psychological support.^8^
- Launch of a free search engine for medical entities, in which communication in Ukrainian, Russian, or English language is possible. The search engine enables automatic location on a map and indicates the nearest units. One can also enter a specific city or address.^8^,^9^
- Glossaries and medical translators.

In the first days of the war, the Naczelna Izba Lekarska (ang. Supreme Medical Chamber) prepared translations of medical interviews and glossaries for medical staff to communicate with refugees from Ukraine.^10^

## Discussion

The war and humanitarian crisis in Ukraine brought about a significant exodus of the inhabitants of Ukraine mainly to Poland. The armed conflict in Ukraine and the wave of refugees flowing into Poland pose a particular challenge for the Polish public health system in terms of providing these people with medical, social, and economic care. Medical assistance in response to health threats accompanying mass migrations, organization of education, labor market, and risks related to the destruction of the migrants' social lives and environment are some of the problems and challenges related to the humanitarian catastrophe caused by war; the effects of which are being and will also be borne by Poland.

The implementation of each of the abovementioned tasks related to medical assistance requires an efficient system for monitoring incoming refugees to identify their needs, including medical needs. Thanks to this, it will be possible, among other things, to direct those in need of help to the appropriate medical centers and to implement appropriate prophylactic, diagnostic, and treatment processes, which in the long run, will have a positive impact on both the assisted and interests related to the health security policy of Poland.^11^ Detailed knowledge on this subject will also allow for effective management of limited medical resources, positively influencing their availability, as well as allowing for deliberate actions, i.e., directly resulting from the health needs of migrants in this area. The current situation is unique, both in terms of the scale of refugees and the assistance provided.

It is important, however, that every citizen of Ukraine in Poland benefits from free hospital care, specialist treatment, dental services, outpatient treatment or first aid from a family doctor, and diagnostic tests (as directed by a doctor). Services provided to Ukrainian citizens are settled by the Polish healthcare payer, the National Health Fund. The situation remains very dynamic, and the range of needs will evolve over time.

## Author Contributions

EF, MM-W, and SD-G analyzed the data, did critical discussion and reading, and wrote the paper. All authors contributed to the article and approved the submitted version.

## Funding

This work was supported by the Grant of Medical University of Silesia in Katowice, Poland.

## Conflict of Interest

The authors declare that the research was conducted in the absence of any commercial or financial relationships that could be construed as a potential conflict of interest.

## Publisher's Note

All claims expressed in this article are solely those of the authors and do not necessarily represent those of their affiliated organizations, or those of the publisher, the editors and the reviewers. Any product that may be evaluated in this article, or claim that may be made by its manufacturer, is not guaranteed or endorsed by the publisher.

## Footnotes

^1^Available online at: https://www.gov.pl/web/mswia-en/the-act-on-assistance-to-citizens-of-ukraine-in-connection-/Dz.U.2022,poz.583/

^2^Available online at: https://coronavirus.jhu.edu/region/ukraine

^3^European Centre for Disease Prevention and Control. *Operational public health considerations for the prevention and control of infectious diseases in the context of Russia's aggression toward Ukraine*. Stockholm (2022).

^4^Available online at: www.szczepienia.pzh.gov.pl

^5^Available online at: https://www.stjude.org/inspire/news/evacuating-children-with-cancer-ukraine.html

^6^Available online at: https://www.gov.pl/web/zdrowie/pomoc-medyczna-dla-ukrainy/; https://www.gov.pl/web/health/medical-help-for-Ukraine

^7^Available online at: https://www.civitas.edu.pl/en/our-university/support-measures/medical-and-psychological-help

^8^Available online at: https://likar.mz.gov.pl

^9^Available online at: www.medicalhelp.pl

^10^Available online at: https://notesfrompoland.com/2022/03/17/ukrainian-refugee-medics-could-boost-polands-understaffed-health-system/

^11^Available online at: https://www.termedia.pl/mz/Jak-przygotowac-sie-do-pomocy-Ukrainie/
